# Altered expression of DNA methyltransferases and methylation status of the TLR4 and TNF-α promoters in COVID-19

**DOI:** 10.1007/s00705-023-05722-9

**Published:** 2023-02-25

**Authors:** Sohair Salem, Rehab Mosaad, Randa Lotfy, Mohamed Elbadry

**Affiliations:** 1 Molecular Genetics and Enzymology Department, Human Genetics and Genome Research Institute, National Research Centre, El Buhouth St., Dokki., P.O. 12622, Cairo, Egypt; 2 Endemic Medicine Department, Faculty of Medicine, Helwan University, Cairo, Egypt

## Abstract

Epigenetic modifications play a significant role in the host's immune response to viral infection. Two epigenetic events, DNA methylation and histone acetylation, are crucial for modifying the chromatin architecture and the location of regulatory elements such as promoters and enhancers. In this case-control study, we evaluated the expression of genes involved in epigenetic machinery (DNMT1, DNMT3A, DNMT3B, HDAC2, and HDAC3) and the degree of methylation of promoters of immune response genes (IFITM1/2/3, TLR3/4, TNF-α, NF-κB, and MYD88) as well as global methylation (LINE-1 and global 5–mC) in blood samples from 120 COVID-19 patients (30 mild, 30 moderate, 30 severe, and 30 critical) and 30 healthy subjects without COVID-19. In contrast to previous reports, DNMT3A and DNMT3B expression was found to be significantly downregulated in COVID-19 cases, whereas DNMT1, HDAC2, and HDAC3 expression did not change. DNMT1 and DNMT3A were negatively correlated with COVID-19 severity. Critically ill patients had lower HDAC3 expression levels. TLR4 and TNF-α had increased promoter methylation, whereas IFITM1/2/3, TLR3, NF-κB, MYD88, and LINE-1 did not differ between cases and controls. Methylation of the TNF-α promoter increased as disease severity increased. Significantly less methylation of the TLR3 promoter was observed in patients with a positive outcome (recovery). We also found a correlation between the expression of DNMT3B and the methylation level of the TLR4 promoter. In milder cases, the global 5–mC levels were lower than that in more severe cases. Our findings suggest the exclusion of DNMTs inhibitors previously recommended for COVID-19 treatment and the need for additional research in this area.

**Supplementary Information:**

The online version contains supplementary material available at 10.1007/s00705-023-05722-9.

## Introduction

Coronavirus disease 2019 (COVID-19) is caused by infection with severe acute respiratory syndrome coronavirus 2 (SARS-CoV-2). It has resulted in the deaths of more than 5 million individuals, with fatality rates of approximately 1% in unvaccinated populations [21]. Several risk factors are associated with disease severity and prognosis across a spectrum of symptoms ranging from mild to life-threatening [20]. Forty percent of cases exhibit silent infection, 40% exhibit benign upper respiratory tract disease, and 20% of the patients in unvaccinated populations suffer from pneumonia [21].

For successful infection, respiratory viruses need to overcome the host immune response through a complex combination of interactions that affect disease outcome [9] and manipulate host gene expression to ensure virus replication [17]. This is achieved in part by altering epigenetic mechanisms of the host, such as DNA methylation and histone modification. Methylation and acetylation are crucial for modifying the chromatin architecture and the positions of regulatory elements such as promoters and enhancers, and they are mediated by DNA methyltransferases (DNMTs) and histone deacetylases (HDACs), whose expression levels vary among different cell types and environmental conditions [4]. During viral infection, epigenetic and transcriptional modifications have been documented in both the virus and the host. The infected host cell induces an antiviral epigenetic response to promote survival pathways, and the virus inhibits the expression of host genes involved in the antiviral response [17]. The epigenetic effector differs between viral infections. For example, it was found that DNA methylation, rather than histone modification, is crucially involved in inhibition of antigen-presentation gene expression mediated by Middle East respiratory syndrome coronavirus (MERS-CoV), whereas a combination of epigenetic mechanisms is used by H5N1-VN1203 influenza virus to target antigen presentation [9]. During infection with SARS-CoV-2, immune cells massively secrete pro-inflammatory cytokines and chemokines, leading to a cytokine storm and acute respiratory distress syndrome [4]. Therefore, it is essential to investigate the epigenetic events involved in regulating the expression of genes related to the immune response.

Long interspersed nuclear element 1 (LINE-1) is one of the major repetitive elements, of which there are over 500,000 copies, representing about 17% of the human genome. LINE-1 is 6 kb long and is composed of a 5´ untranslated region with an internal RNA polymerase II promoter, two open reading frames, and a 3´ UTR with a polyadenylation signal. It has been shown that the degree of methylation of LINE-1 reflects the global level of methylation [6].

In this study, we analyzed the expression profile of DNMTs (DNMT1, DNMT3A, DNMT3B) and HDACs (HDAC2 and HDAC3) in COVID-19 patients. We also examined the degree of promoter methylation for IFITM1/2/3 (interferon-induced transmembrane proteins), TLR3/4 (Toll-like receptors), TNF-α (tumour necrosis factor alpha), NF-κB1 (nuclear factor kappa B subunit 1), and MYD88 (myeloid differentiation primary response 88) in addition to the degree of methylation of LINE-1 as an indicator of global methylation. The global level of 5 methyl cytosine (5-mC) was analyzed using an enzyme-linked immunosorbent assay (ELISA).

## Subjects and methods

### Subjects

One hundred twenty patients with newly confirmed COVID-19 by real-time PCR of nasopharyngeal swabs were enrolled in the study. They were clinically divided into four subgroups based on the WHO case definition. *Mild*: the clinical symptoms were mild, and lung imaging revealed no pneumonia manifestations. *Moderate*: patients exhibited symptoms such as fever and respiratory tract symptoms, and lung imaging revealed no pneumonia manifestations. *Severe*: patients who met any of the following criteria: respiratory rate 30 breaths/min; oxygen saturation < 93% at rest; arterial partial pressure of oxygen (PaO_2_)/fraction of inspired oxygen (FiO_2_) <300 mm Hg. Patients with more than 50% of lesions within 24 to 48 h in lung imaging were also treated as severe cases. *Critical*: patients who met any of the following criteria: respiratory failure requiring mechanical ventilation, shock, or other organ failure requiring monitoring and treatment in the intensive care unit (patient data are shown in Table 1). All cases were evaluated clinically using a specially designed detailed registration sheet, a thorough chest and general examination, and detailed laboratory investigations to detect any potential complications or deterioration until recovery was confirmed by real-time PCR. Patients were recruited between May and August 2021 from Abbasia Fever Hospital, one of the COVID-19 isolation hospitals assigned by the Egyptian Ministry of Health and Population. Patients provided written informed consent to participate, and the study protocol was approved by the ethical research committee of the Ministry of Health and Population (registration number: 8-2021/19). Additionally, the study included 30 individuals without a history of COVID-19 infection or confirmed patient contact, as a control group.

**Table 1:** Characteristics of COVID-19 patients in this study

	Number (%)
Sex	
Male Female	78 (65%) 42 (35%)
Severity	
Mild Moderate Severe Critically ill	30 (25%) 30 (25%) 30 (25%) 30 (25%)
Outcome	
Cure Death	94 (78%) 26 (22%)

### Quantitative real-time PCR (qPCR)

Immediately after the samples were collected, total RNA was extracted from whole blood using Direct-zol RNA Miniprep (Zymo Research) according to the manufacturer's instructions. The extracted RNA was reverse transcribed into single-stranded complementary DNA (cDNA) using a RevertAid First Strand cDNA Synthesis Kit (Thermo Fisher Scientific). For mRNA expression analysis, mixtures were prepared using Maxima SYBR Green qPCR Master Mix (Thermo Fisher Scientific) according to manufacturer's recommendations. GAPDH was used as an internal reference gene to normalize gene expression. Primer sequences of DNMT1, DNMT3A, DNMT3B, HDAC2, and HDAC3 (Supplementary Table S1) were designed using Primer3 software (http://bioinfo.ut.ee/primer3-0.4.0/primer3/). Results were expressed as a ratio of reference to target gene using the 2^-∆Ct^ method.

### Methylation-specific PCR (MSP)

DNA was extracted using a standard salting-out protocol from blood samples stored at -80°C. The extracted DNA was subjected to bisulfite modification using an EpiTect Bisulfite Kit (QIAGEN). The converted DNA was used for methylation-specific PCR (MSP), in which two different sets of primers (methylated and unmethylated) were employed to amplify each gene separately. Each set of MSP reactions was carried out in a 15-µl reaction using 7.5 µl of Maxima SYBR Green Master Mix (Thermo Scientific), 2 µl of treated DNA, and 150 nM of each primer. The percentage of methylation was calculated using the CT method according to the following equation: the percentage of methylation = 100/[1+2^ΔCt(methylated-unmethylated)^] %. The LINE-1 sequence was downloaded from NCBI (https://www.ncbi.nlm.nih.gov/nuccore/L19088.1). Primers for LINE-1 and promoters of IFITM1/2/3, TLR3/4, TNF-α, NF-κB1, and MYD88 were designed using the free online methprimer program (http://www.urogene.org/methprimer), using CpG island prediction for primer selection. Primers were designed to flank CpG islands in the case of small islands or to be located within the island in the case of large islands. When the promoter sequence contained two islands that were located close to each other (NF-κB1 promoter), primers were designed so that the amplicon would span both islands (product lengths and primer sequences are shown in Supplementary Table S1). Duplicate calibrator samples were used in all runs to allow a comparison of the results across all runs.

### ELISA assay for global methylation

A MethylFlash™ Global DNA Methylation (5-mC) ELISA Easy Kit (Colorimetric) was used to determine the percentage of methylated DNA (5-mC%) according to the instructions of the manufacturer.

### Statistical analysis

Data were statistically analyzed using IBM SPSS Statistics (Statistical Package for the Social Sciences) software version 18.0 (IBM Corp., Chicago, USA, 2009). Descriptive statistics were done for quantitative data, which are reported as the mean ± SD. For each assay, all samples were run in duplicate. The independent *t*-test was used to compare levels of expression and methylation between groups, cases vs. healthy subjects, and positive (recovery) vs. negative (death) outcomes, whereas an ANOVA test was used to compare expression and methylation percentage between groups of patients with varying severity (mild, moderate, severe, and critical). To evaluate the utility of using expression and methylation levels as discriminatory biomarkers for COVID-19 patients, a receiver operating characteristic (ROC) curve was used. Binary logistic regression was used to analyze the combined ROC curve*.* The correlation coefficient was used to determine the degree of association between the level of expression and methylation and disease characteristics, severity, and outcome. The significance threshold was set at < 0.05.

## Results

### Altered expression of DNMTs and HDAC3 in COVID-19 patients

**Fig. 1:**
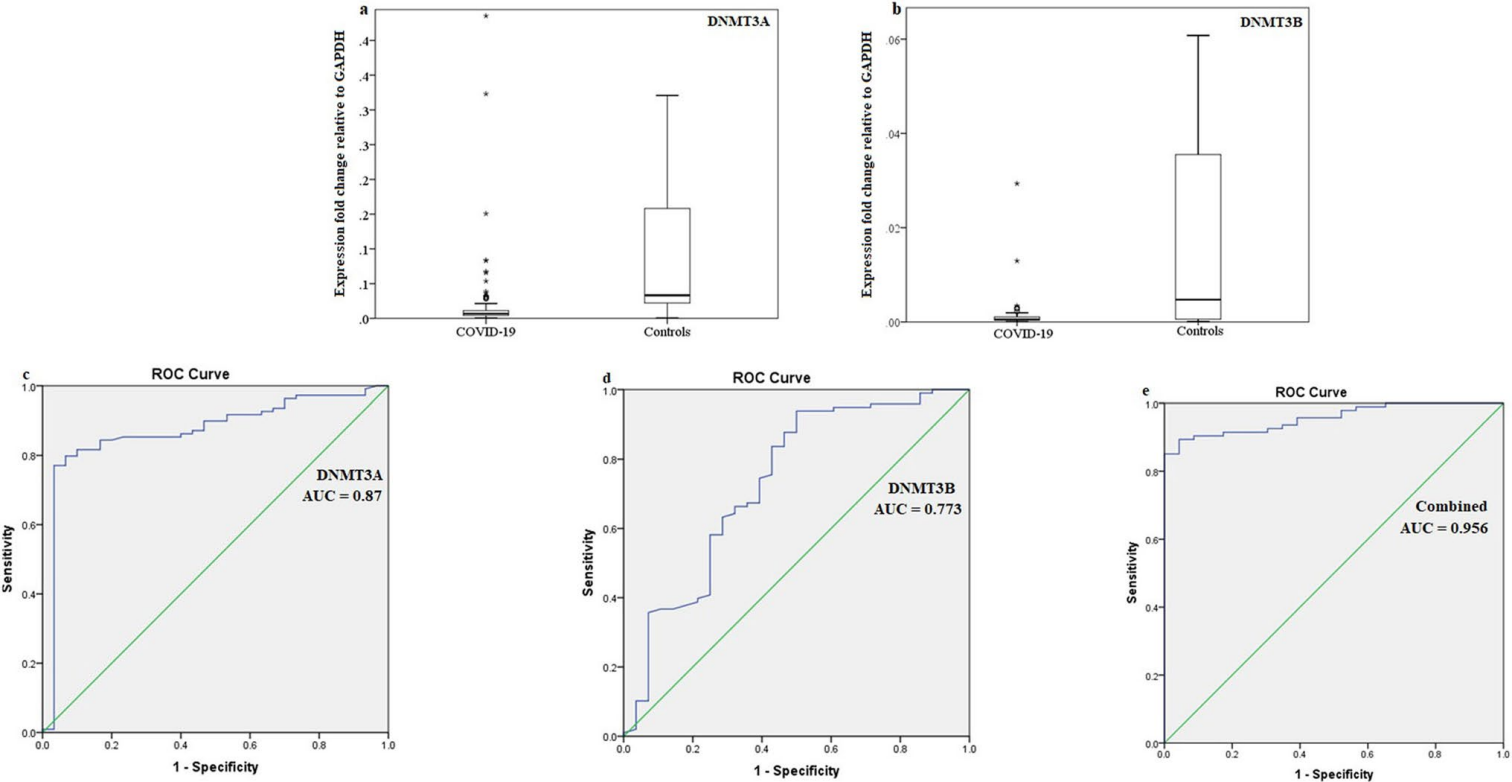
Alteration of DNMT3A and DNMT3B expression in COVID-19 patients. (a) Box plot demonstrating the downregulation of DNMT3A in cases relative to controls. (b) Box plot demonstrating the downregulation of DNMT3B in cases relative to controls. (c) ROC curve for DNMT3A. (d) ROC curve for DNMT3B. (e) ROC curve for DNMT3A and DNMT3B combined

Compared with the healthy COVID-19-free group, the expression of DNMT3A and DNMT3B in the blood of COVID-19 patients was decreased (*P* = 0.008 and 0.048, respectively). The area under the ROC curve (AUC) for each was 0.87 and 0.773, respectively, and the combined AUC was 0.956% (Table 2 and Fig. 1). DNMT1, DNMT3A, and DNMT3B were inversely correlated with COVID-19 (r = -0.215, -0.528, and -0.335, respectively). DNMT1 and DNMT3A had an inverse correlation with the severity of COVID-19 (r = -0.242 and -0.233, respectively; Table 3). Expression levels of DNMT1, DNMT3A, and DNMT3B were positively correlated with each other (Table 4). HDAC3 was differentially expressed among the severity groups and was found to be downregulated in critically ill patients (*P* = 0.005) (Table 5).

**Table 2:** Comparison of DNMTs expression in COVID-19 cases and controls

	Independent *t*-test		ROC curve	
	Mean fold difference in expression (± SD)	*P*	AUC	*P*
DNMT3A				
Patients Controls	0.02 ( *±* 0.07) 0.11 ( *±* 0.16)	0.008	0.87	<0.001
DNMT3B				
Patients Controls	0.009 ( *±* 0.04) 0.08 ( *±* 0.17)	0.048	0.773	<0.001
Combined			0.956	<0.001

The fold expression change was determined by qPCR and calculated using the 2^-∆Ct^ formula relative to GAPDH as a reference gene. *P* is significant at a level of <0.05. AUC, area under the ROC curve

**Table 3:** Correlation between DNMTs and COVID-19 disease and its severity

Gene	Disease		Severity	
	r	*P*	R	*P*
DNMT1	**-0.215**	**0.009**	**-0.242**	**0.01**
DNMT3A	**-0.528**	**<0.001**	**-0.233**	**0.015**
DNMT3B	**-0.335**	**<0.001**	0.037	0.72

*P* is significant at a level of <0.05. r, Spearman correlation coefficient

**Table 4:** Correlation between DNMTs expression, and promoter methylation for IFITM1, IFITM2, IFITM3, TNF*-*α, TLR4, MYD88, and LINE-1 in COVID-19 patients

	r	*P*
DNMT1 and DNMT3A	0.424	<0.001
DNMT1 and DNMT3B	0.905	<0.001
DNMT3A and DNMT3B	0.873	<0.001
IFITM1 and TNF-α	0.245	0.024
IFITM1 and LINE-1	0.209	0.047
IFITM2 and LINE-1	0.214	0.037
IFITM3 and TNF-α	0.236	0.027
TLR4 and MYD88	0.221	0.042
TLR4 and LINE-1	0.279	0.002

*P* is significant at a level of <0.05. r, Pearson correlation coefficient

**Table 5:** Expression fold of HDAC3 in different severity groups of COVID-19 patients

	ANOVA		Spearman correlation	
	Mean difference in fold expression (± SD)	*P*	r	*P*
Mild	.023 (.032)			
Moderate	.012 (.009)	0.005	-0.455	<0.001
Severe	.01 (.008)			
Critical	.007 (.004)			

The fold expression change was determined by qPCR and calculated using the 2^-∆Ct^ formula relative to GAPDH as a reference gene. *P* is significant at a level of <0.05. r, Spearman correlation coefficient, which revealed a negative correlation between HDAC3 expression and COVD-19 severity

### Increased promoter methylation of TLR4 and TNF-α in COVID-19 patients

**Fig. 2:**
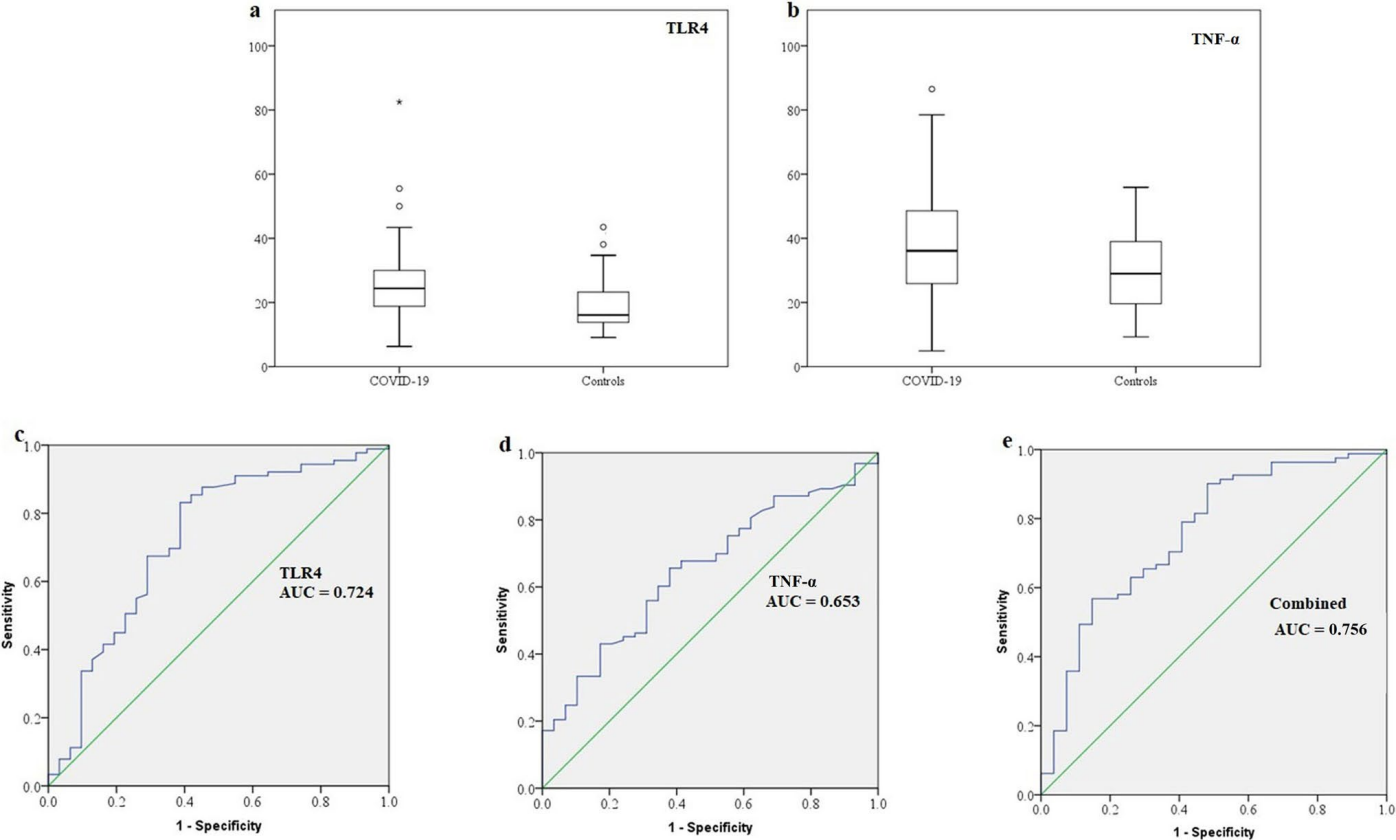
Alteration of TLR4 and TNF-α promoter methylation in COVID-19 patients. (a) Box plot depicting a higher percentage of methylation of the TLR4 promoter in cases compared to controls. (b) Box plot displaying a higher percentage of methylation of the TNF-α promoter in cases than in controls. (c) ROC curve for TLR4. (d) ROC curve for TNF-α. (e) ROC curve for TLR4 and TNF-α combined

The percentage of methylation of the TLR4 and TNF-α promoters was significantly higher in COVID-19 patients than in healthy subjects (*P* = 0.003 and 0.005, respectively). Analysis of the ROC curve showed that the AUC for methylation of TLR4, TNF-α, and both was 0.724, 0.653, and 0.756, respectively (Table 6 and Fig. 2). IFITM1/2/3, TLR3, NF-κB1, and MYD88 showed no significant difference between cases and controls (*P* = 0.291, 0.515, 0.43, 0.79, 0.614, and 0.169, respectively) (Supplementary Fig. S1).

**Table 6:** Percentage of TLR4 and TNF-α promoter methylation in COVID-19 patients and controls

	Independent *t*-test		ROC curve		Spearman correlation	
	Mean percentage of methylation (± SD)	*P*	AUC	*P*	r	*P*
TLR4					0.339	<0.001
Patients	25.6 (10.5)	0.003	0.724	<0.001		
Controls	19.3 (8.3)					
TNF-α			0.653	0.013	0.225	0.013
Patients	38.3 (17)	0.005				
Controls	29.6 (12.5)					
Combined			0.756	<0.001		

*P* is significant at a level of <0.05. AUC, area under the ROC curve; r, correlation coefficient

The percentage of methylation of the TNF-α promoter increased significantly with increasing severity: 29.6%, 43.6%, 37.6%, and 41.7% for mild, moderate, severe, and critical cases, respectively, *P* = 0.025 (Table 7). Our data also showed that patients with a positive outcome (recovery) had significantly less methylation of the TLR3 promoter (54%) than patients with a negative outcome (death), who had a significantly higher level of methylation (66%) (Table 8). A positive outcome was also correlated with the percentage of LINE-1 methylation (r = 0.202, *P* = 0.043). Additionally, there was a significant correlation between DNMT3B expression and the degree of TLR4 methylation (r = 0.27 and *P* = 0.019). The promoter methylation values for IFITM1, IFITM2, IFITM3, TNF-α, TLR4, MYD88, and LINE-1 were correlated with each other, as shown in Table 4.

**Table 7:** Methylation of the TNF-α promoter in different severity groups of COVID-19 patients

Severity group	Mean percentage of methylation (± SD)	*P*	95% CI
Mild	29.6 (14.7)		23.27 – 36
Moderate	43.6 (18.2)	0.025	35.7 – 51.45
Severe	37.6 (15.7)		30.4 – 44.7
Critical	41.7 (17)		34.86 – 48.6

*P* was calculated by ANOVA.

**Table 8:** Comparison of TLR3 promoter methylation and global 5-mC between COVID-19 patients with different severity and outcome

	Percentage of methylation		Spearman correlation	
	Mean (± SD)	*P*	r	*P*
*TLR3 promoter*				
**Outcome**				
Recovery	54 (17)	0.002	0.304	0.003
Death	66 (14.2)			
*Global 5 mC*				
**Severity**				
Mild	2.9 (1.5)			
Moderate	4.4 (2.9)	<0.001	0.482	<0.001
Severe	5.4 (2.2)			
Critical	5.8 (1.5)			
**Outcome**				
Recovery	4.2 (2.1)	0.004	0.253	0.005
Death	5.6 (2.2)			

The significance of mean values was calculated by independent *t*-test in the case of “outcome” and by ANOVA in the case of “severity”. *P* is significant at a level of <0.05. r, correlation coefficient

### Association of global 5-mC with COVID-19 severity and outcome

There was no statistical difference between cases (4.6% [±2.2%]) and controls (5.4% [±1.9]), in terms of global 5-mC (*P* = 0.078), but there was a significant difference between the severity groups, where the percentage of global 5-mC was 2.9, 4.4, 5.4, and 5.8 for mild, moderate, severe, and critical cases, respectively. Additionally, significantly less global 5-mC was observed in patients with a positive outcome (4.2%) than in those with a negative outcome (5.6%) (Table 8).

## Discussion

Understanding the epigenetic profile could yield potential therapeutic targets that serve as markers of disease severity and prognosis. The main epigenetic events, DNA methylation and histone acetylation, are mediated by DNMTs and HDACs and are crucial for regulating chromatin packaging and the positioning of regulatory elements [4]. In response to enhanced viral replication, these epigenetic events may be involved in long-term infection, clinical phenotypes of severity, and fatality in COVID-19 cases [7]. DNMTs and HDACs are expressed differentially in various cell types. We conducted a case-control study to investigate the differential expression of DNMT1, DNMT3A, DNMT3B, HDAC2, and HDAC3 in the blood of 120 patients (30 mild, 30 moderate, 30 severe, and 30 critical) and 30 healthy subjects without COVID-19.

DNMT1 acts on hemimethylated DNA and plays an important role during replication, while DNMT3A and DNMT3B act on unmethylated DNA to mediate *de novo* methylation during development [3]. Elevated DNMTs have been reported to be involved in regulation of viral latency. Furthermore, DNMT inhibitors are thought to inhibit DNA methylation in macrophages and control coronavirus infection [14]. In our study, COVID-19 patients displayed unexpected significant downregulation of DNMT3A and DNMT3B expression. Analysis of an ROC curve identified both DNMT3A and DNMT3B as predictive biomarkers for COVID-19, and their combination increases their predictive power, as indicated by increasing AUC. A previous study revealed a decreased abundance of DNMT1, DNMT3A, and DNMT3B in lung epithelial cell lines infected with SARS-CoV-2; however, this decrease was not observed in lung tissues from a COVID-19 patient [12]. In our study, there was no difference in the expression of DNMT1 between cases and controls. A study on hepatitis B virus (HBV) revealed that DNMTs are elevated after productive viral replication due to the immune response mechanism of the host [7]. In our study, all DNMTs were inversely correlated with COVID-19 disease, whereas DNMT1 and DNMT3A, but not DNMT3B, had an inverse relationship with disease severity. It was found that the oxidative stress induced by SARS-CoV-2 infection led to inhibition of DNMT1 and alteration of DNA methylation [18]. In addition, we found that DNMT1, DNMT3A, and DNMT3B expression were positively correlated. Numerous diseases, such as heart disease [19] and systemic lupus erythematosus [1], have been examined to determine the correlation between DNMTs, which were found to be directly correlated.

It has been reported that HDAC2 regulates the activity of NF-κB and alters monocyte and macrophage function, making it crucial for the immune evasion strategy of SARS-CoV-2 [4]. Inhibition of HDAC3 has been shown to induce anti-inflammatory activity, reduce pro-inflammatory cytokine production, and block IL-6 and TNF-α production. An *in vitro* study suggested a potential role for HDAC inhibitors in reducing the release of cytokines by epithelial cells, monocytes, and macrophages. These inhibitors also decrease the activation of monocytes and enhance the differentiation of T cells. A previous study demonstrated the ability of the HDAC inhibitors to hinder IFN-1 expression and downstream effects [16]. HDAC inhibitors have been found to regulate SARS-CoV-2 entry in epithelial cells by decreasing the expression of angiotensin converting enzyme 2 (ACE2) and blocking ACE2-mediated entry of the virus; consequently, they have been proposed as potential therapeutic agents for COVID-19 [16]. In our study, there was no significant difference in the expression of HDAC2 and HDAC3 between COVID-19 patients and the control group; however, HDAC3 was associated with severity, with lower expression levels correlating with more-severe disease. Our results suggest that further research is needed before recommending DNMT and HDAC inhibitors as therapeutic drugs for COVID-19.

The change in the expression of DNMTs led us to hypothesize a shift in the methylation pattern of immune response gene promoters. We examined the methylation of the promoters of IFITM1/2/3, TLR3/4, TNF-α, NF-κB1, and MYD88, in addition to LINE-1 methylation.

Compared to healthy subjects, we found that only the promoters of TLR4 and TNF-α exhibited altered methylation patterns. Considering that expression of TNF-α [5, 7] and TLR4 [10] has been reported to be elevated in COVID-19 patients and that DNA methylation is associated with decreased gene expression, we predicted that methylation of these promoters would be decreased; however, our results demonstrated an increase in the percentage of methylation of the promoters of both genes in COVID-19 patients, and the severity of disease was associated with a higher percentage of methylation of the TNF-α promoter. This could be explained by the previously described positive association between DNA methylation and gene expression. Some transcription factors preferentially bind to methylated CpG over unmethylated CpG. A reduction in 5-mC residues in a promoter has the ability to hinder the binding of transcription machinery although the region as a whole is methylated. Local methylation of individual residues may upregulate gene expression and counteract the methylation pattern of the genomic region as a whole [15]. We adopted this explanation for TNF-α for two reasons: first, the overall low percentage of methylation in all subjects of our study (although the percentage was significantly higher in cases than in controls), and second, the reduced level of DNMT3B mRNA detected in our study. A previous study indicated that TNF-α stimulation leads to suppression of ACE2 expression in endothelial cells and enhancement of DNA methylation in the ACE promoter via DNMT3A and DNMT3B, but not DNMT1 [11]. That study showed that stimulation of TNF-α induced a transient decrease in DNMT3B and TET1 (translocation methylcytosine dioxygenase) as a mediator of intermediate steps to remove the methyl group from 5’-methylcytosine. It also showed that downregulation of DNMT3A and DNMT3B lessened the effect of TNF-α on ACE [11]. In the case of TLR4, we found a positive correlation between DNMT3B expression and methylation of the TLR4 promoter, suggesting the involvement of DNMT3B in mediating TLR4 methylation.

We also found that patients with poor outcome (death) had elevated TLR3 promoter methylation. According to a previous report [10], TLR3 expression is reduced in the peripheral blood of COVID-19 patients, and this reduction is associated with unfavourable outcome.

Global hypomethylation may reflect genomic instability [13]. For the analysis of global DNA methylation, we measured the percentage of methylation of LINE-1, using MSP, and global 5-mC, using ELISA. LINE-1 hypomethylation has been reported to be associated with an increase in its transcriptional activation and expression, whereas methylation of LINE-1 within the CpG-rich 5′-UTR inhibits its activation and transcription, thereby reducing the DNA damage resulting from its hypomethylation [6]. In our study, there was no change in the percentage of methylation of LINE-1 in COVID-19 patients, nor was there a correlation between it and the severity of the disease; however, it positively correlated with favourable outcome. Using a specific ELISA, we found that global DNA 5-mC levels did not differ between cases and controls. Unexpectedly, we found a significantly lower level of global methylation in mild versus severe cases and in patients with a positive outcome versus those with a negative outcome. A previous study in which genome-wide analysis of CpG methylation in blood of COVID-19 patients was carried out revealed no difference in the mean global methylation between patients and healthy subjects [2]. However, 13,033 differentially methylated sites were identified in a case-control epigenome-wide association study [8].

## Conclusion

The study revealed a pattern of decreased expression of DNMT genes (DNMT3A and DNMT3B) and an increase in the methylation of the TNF-α and TLR4 promoters in COVID-19 patients, as well as a correlation between global methylation and disease severity. The reduced DNMT expression indicates the need for more research before recommending the use of DNMT inhibitors for COVID-19.

## Supplementary Information

Below is the link to the electronic supplementary material.

Supplementary file1 Supplementary Fig. S1 Absence of alteration in promoter methylation levels for IFITM1/2/3, TLR3, NF-κB1, and MYD88 in COVID-19 patients compared to control subjects (JPG 71 KB)

Supplementary file2 (DOCX 14 KB)

## Data Availability

No data were associated with this manuscript.
