# Supplementary figures and images for: Altered expression of DNA methyltransferases and methylation status of the TLR4 and TNF-α promoters in COVID-19
Source: Arch Virol. 2023 Feb 25;168(3):95. doi: 10.1007/s00705-023-05722-9 (PMC9959945; doi:10.1007/s00705-023-05722-9)

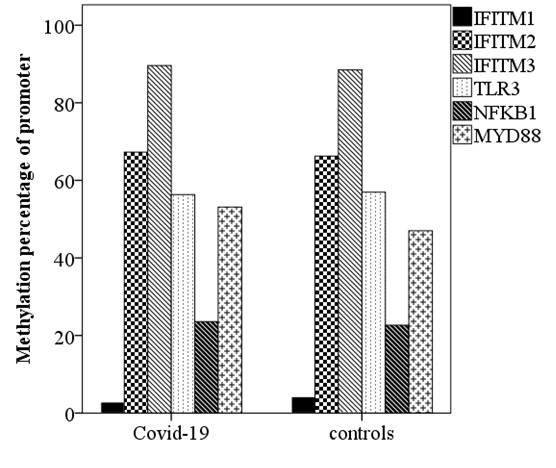

Supplement: Supplementary file 1 — Supplementary file1 Supplementary Fig. S1 Absence of alteration in promoter methylation levels for IFITM1/2/3, TLR3, NF-κB1, and MYD88 in COVID-19 patients compared to control subjects (JPG 71 KB) [file 705_2023_5722_MOESM1_ESM.jpg]
