# Supplementary material for: Altered expression of DNA methyltransferases and methylation status of the TLR4 and TNF-α promoters in COVID-19
Source: Arch Virol. 2023 Feb 25;168(3):95. doi: 10.1007/s00705-023-05722-9 (PMC9959945; doi:10.1007/s00705-023-05722-9)
Supplement: Supplementary file 2 — Supplementary file2 (DOCX 14 KB) [file 705_2023_5722_MOESM2_ESM.docx]

Supplementary Table 1: Sequence of primers used for qPCR and MSP.

| Gene | Sequence (5 → 3) | Product size  (bp) |
| --- | --- | --- |
| HDAC2- F | CTTGGCAAGTTTTATTGTGAGTTTT | 166 |
| HDAC2-R | TTACAAAAGATGGAAAAATCAGCTC |  |
| HDAC3-F | GTTCAAGAAGCTTTCTACCTCACTG | 214 |
| HDAC3-R | GGTAGAAGTCCACTACCTGGTTGAT |  |
| DNMT1-F | GAGAACACCCACAAGTCCACTC | 187 |
| DNMT1-R | CGAGGAAGTAGAAGCGGTTG |  |
| DNMT3A-F | TTTTAACTCTCGCCTCCAAAGAC | 141 |
| DNMT3A-R | AGAGCTGCTGGTGTCCCC |  |
| DNMT3B-F | GCCGGCTCTTCTTCGAATTT | 140 |
| DNMT3B-R | CTCCAGGAACCGTGAGATGT |  |
| LINE-1-F-M | GAGGTATTGTTTTATTTGGGAAGC | 209 |
| LINE-1-R-M | TACTAACAATCAACGAAATTCCGTA |  |
| LINE-1-F-U | AGGTATTGTTTTATTTGGGAAGTGT | 210 |
| LINE-1-R- U | TACTAACAATCAACAAAATTCCATA |  |
| TLR3-F-M | ATAGTGTTGTAAAGAGATAAAAGTTAGACG | 197 |
| TLR3-R-M | CCTCACCATTTAATATAAACGAAT |  |
| TLR3-F-U | AGTGTTGTAAAGAGATAAAAGTTAGAT | 199 |
| TLR3-R-U | AAAACCTCACCATTTAATATAAACAAA |  |
| TLR4-F-M | TTTTTTATAAGAAGGGGCGGGT | 385 |
| TLR4-R-M | AACCCTTATAACTCTCTAAAATAACGAT |  |
| TLR4-F-U | TTTTTATAAGAAGGGGTGGGT | 385 |
| TLR4-R-U | TAACCCTTATAACTCTCTAAAATAACAA |  |
| MYD88-F-M | ATTTCGAGGGTAGGGTTACGGT | 420 |
| MYD88-R-M | CCCTTAAAATCTCGAAACGAAT |  |
| MYD88-F-U | TTTGAGGGTAGGGTTATGGTA | 420 |
| MYD88-R-U | TACCCTTAAAATCTCAAAACAAAT |  |
| NF-κB1-F-M | ATTGATTGGGTTCGGTAGGCGT | 416 |
| NF-κB1-R-M | GAACCGCTCCGATAACGAAA |  |
| NF-κB1-F-U | GATTGGGTTTGGTAGGTGT | 415 |
| NF-κB1-R-U | CCAAACCACTCCAATAACAAAA |  |
| TNF-α-F-M | TTTAGTTTCGATTTAGAATTCGTTC | 115 |
| TNF-α-R-M | AAAAAAACCTCACCTACTATACGAA |  |
| TNF-α-F-U | TTAGTTTTGATTTAGAATTTGTTTGT | 119 |
| TNF-α-R-U | AAAACAAAAAAACCTCACCTACTATACA |  |
| IFITM1-F-M | ATGTGGGGTGTTTTATGGGC | 416 |
| IFITM1-R-M | GACGCTACGAATCTCAAAATCC |  |
| IFITM1-F-U | GTGGGGTGTTTTATGGGTGG | 418 |
| IFITM1-R-U | ATCCAACACTACAAATCTCAAAATCC |  |
| IFITM2-F-M | AATTTTGGTTTATTGTAAGTTTCGT | 152 |
| IFITM2-R-M | CTAACTAACACGATAAAACCCCGT |  |
| IFITM2-F-U | ATTTTGGTTTATTGTAAGTTTTGT | 151 |
| IFITM2-R-U | CTAACTAACACAATAAAACCCCATC |  |
| IFITM3-F-M | GTATTATAGTGAGGGTTATGGGAGACGG | 421 |
| IFITM3-R-M | TATCAAATCGAAATTTAACCGAAA |  |
| IFITM3-F-U | TTATAGTGAGGGTTATGGGAGAT | 422 |
| IFITM3-R-U | CATTTATCAAATCAAAATTTAACCAAAA |  |

^F: forward; R: reverse; M: methylated; U: unmethylated; bp: base pair.^
